# Treating Parental Burnout: Impact and Particularities of a Mindfulness- and Compassion-Based Approach

**DOI:** 10.3390/children11020168

**Published:** 2024-01-27

**Authors:** Marie Bayot, Maria Elena Brianda, Nastasya van der Straten, Moïra Mikolajczak, Rebecca Shankland, Isabelle Roskam

**Affiliations:** 1 Department of Clinical Sciences, University of Liège, CHU du Sart Tilman–Quartier Hôpital–B23, Avenue Hippocrate 13, 4000 Liège, Belgium; marie.bayot@uliege.be; 2 Psychological Research Institute, Catholic University of Louvain, Place Cardinal Mercier 10, 1348 Louvain-la-Neuve, Belgiummoira.mikolajczak@uclouvain.be (M.M.); 3 Psychological Research Institute, University of Liège, Quartier Agora–B33, Place des Orateurs 1, 4000 Liège, Belgium; mariaelena.brianda@uliege.be; 4 Département de Psychologie du Développement, de l’Éducation et des Vulnérabilités, Université Lumière Lyon 2, Avenue Pierre Mendès-France 5, 69676 Bron, France; rebecca.shankland@univ-lyon2.fr

**Keywords:** parental violence, parental neglect, mindfulness-based programs, mindful parenting

## Abstract

Mindfulness- and self-compassion-based programs have been shown to reduce parental stress, and levels of mindfulness and self-compassion have been shown to be negatively related to parental burnout (PB) factors. Based on these results, the present study aimed to test the efficacy of an 8-week mindfulness and compassion-based group approach (MCA) (*n* = 29) compared with the existing Parenting in Balance Program (PBP) (*n* = 25). Parents were blindly enrolled in one of the two conditions. Parental burnout, parental neglect and violence, irritability, parental balance between stress-enhancing and stress-alleviating factors, hair cortisol, and mindful parenting and self-compassion were measured before, after, and three months after the end of the program. All the measured outcomes positively changed over time in both conditions, except for irritability. Large effect sizes were found for parental burnout, parental neglect and violence, and mindful parenting and self-compassion. However, contrary to our hypothesis, the decrease in parental burnout in the MCA was not significantly related to an increase in mindful parenting nor self-compassion. Furthermore, certain participants from the MCA group reported higher levels of parental burnout after the intervention. The absence of specific effects between MCA and PBP programs suggests the presence of common effectiveness factors. Therefore, future studies need to analyze specific variables that may explain differential effects of programs on parental burnout levels.

## 1. Introduction

Parental burnout (PB) reflects a three-dimensional stress syndrome experienced by up to 8% of parents in Western countries [1], encompassing: (1) an overwhelming exhaustion related to one’s parental role; (2) emotional distancing from one’s child(ren); and (3) feelings of being fed up with one’s parental role, which all contrast with the previous parental self [2]. Like job burnout, PB results from a chronic imbalance between stress-enhancing factors (e.g., family disorganization, parenting role restriction, parental perfectionism, neuroticism, dysfunctional coping strategies) and stress-alleviating factors (e.g., coparental support, parenting skills, parenting self-efficacy, emotional intelligence, family climate, shared time between parent and child) [3,4,5,6,7]. The Balance between Risks and Resources scale (BR^2^) [3], composed of bipolar items assessing these factors (i.e., a positive score reflecting heavier resources than risks), indeed shows a strong linear relationship with PB symptoms’ severity and therefore offers a relevant framework to predict and explain PB. Parental stress—parents’ perceived stress associated with the demands of parenting—can therefore be considered as a precursor of PB, which, if chronically uncompensated by parental-stress-alleviating factors, can lead to exhaustion and loss of meaning regarding the parental role. After a prolonged period of acute parental stress, burned-out parents undergo a radical change in their behavior (i.e., irritable, negligent) and self-image (i.e., failure). Because of insufficient resources, they find themselves beyond the point of actively looking for solutions to challenges in child rearing, and tend to experience helplessness. The COVID-19 pandemic is one example of a situation that highly increased parental stress and risk/resource imbalances, especially in parents with social and economical difficulties, who lacked sufficient support and protection factors such as self-compassion [8,9,10,11,12,13,14,15,16]. 

PB has deleterious consequences for the parent (e.g., escape suicidal ideations, health problems), the couple (e.g., increased frequency and intensity of conflicts), as well as the child(ren) (e.g., neglectful and violent behaviors) [17]. While the effect of PB on the parent is comparable in scale to that of job burnout and depression, its effect on neglectful and violent behaviors against the child(ren) is significantly larger than that of job burnout and depression [18].

Although researchers have already suggested that parenting can lead to exhaustion to such a degree that it meets the criteria for burnout [19], the existence of parental burnout (PB) as a specific syndrome distinct from parental stress, job burnout, and depression has only recently been demonstrated [18]. Corollary, interventions to treat PB are still being developed and tested in an attempt to decrease PB and its negative effects on parents and children [20]. Pioneering work by Lindström et al. [21], as well as Brianda et al. [22], showed that 8- to 12-week group-based interventions could significantly reduce burnout levels. Coming from several years of investigation on PB symptoms, assessment, and correlates, Brianda and colleagues identified two potential group treatment approaches: “Directive” and “Non-directive”. The former consisted of actively restoring the balance between stressors and resources or stress-alleviating factors that weigh the most on parenting balance, namely, psychological characteristics of the parent (e.g., perfectionism, emotional competencies), child-rearing practices (e.g., parenting role restriction, autonomy demands), and family functioning (e.g., family disorganization, support from the coparent) [3,23]. These defining features of PB were addressed via psychoeducation and targeted exercises. The non-directive approach consisted of a setting in which parents could be heard and understood without judgment, inspired by experiential support groups. Group support and unconditional positive regard offered to parents aimed at replenishing their capacity to find their own resources and ways out of PB. Importantly, both interventions led to a significant reduction in hair cortisol (i.e., an objective measure of chronic stress; Stalder et al., 2017 [24]) (Cohen’s *d* = 0.53), parental neglect (Cohen’s *d* = 0.43 f = 0.22), and violence (Cohen’s *d* = 0.58). Interestingly, qualitative feedback from both instructors and participants put forth the potential complementarity between the two approaches. While the flexibility in rhythm and content found in the non-directive approach allowed for tailoring to participants’ momentary needs from one session to another, the concrete tools and home practices provided in the directive approach were reassuring for participants and came out as powerful drivers of change. As a result, Brianda and colleagues merged the two approaches into a hybrid program that has yet to be tested, called the Parenting in Balance Program (PBP) [25]. To date, this program is the only tailor-made treatment for burnout in the parental sphere available in the literature.

Looking at other approaches, mindfulness—a way of gently directing one’s attention to present moment experience without judgment—is related to positive outcomes for parents and children. As a matter of fact, mindful parenting has been associated with parents’ mental health (e.g., reducing depression and anxiety scores), as well as parental cognition (e.g., greater perceptions of parental competence, attenuated perception of parenting daily hassles, child perspective taking) and behaviors (e.g., child-oriented solicitation), that influence children’s behavior (e.g., disclosure) and well-being (e.g., emotion regulation) [26,27,28]. As argued by several authors, the unbiased, open-minded attention to thoughts and sensations helps parents to disengage from maladaptive cognitions, hence fostering emotion regulation, and thus facilitates deliberate endorsement of valued parenting practices [29,30]. More specifically, several processes involved in the practice of mindfulness seem to be particularly relevant for the treatment of PB, as they compete with dysfunctional processes that characterize this syndrome, such as struggling with (vs. accepting) unescapable situations [31] or autopilot mode [2]. Autopilot, which illustrates the emotional distancing dimension of PB, consists of acting while barely paying attention to one’s direct environment and one’s own experience (e.g., our emotions, physical sensations). This mode is a matter of survival for burned-out parents who function on depleted batteries. It allows them to continue providing basic care to their children (e.g., driving to school, cooking) without being overwhelmed by one’s and others’ negative emotions. Yet, the autopilot mode sustains the problem by deafening burned-out parents to their suffering and cries for help, as well as increasing the feeling of distance from their child (which in turn deteriorates parents’ self-image). On the contrary, practicing mindfulness fosters emotion regulation and awareness of one’s personal limits and needs in a caring way [32]. Therefore, mindfulness may help burned-out parents to choose to act in ways that promote stress reduction (e.g., help seeking) and positive relationships with family members (e.g., non-violent communication).

As a core component of mindfulness, self-compassion—characterized by an understanding and tender attitude towards one’s own difficulties—might also act as an antidote to PB. As a matter of fact, it has been shown to reduce self-criticism, rumination on negative events and personal flaws, and feelings of shame and guilt [33]. As a result, self-compassion acts on common antecedents of PB, such as dysfunctional perfectionism [34], and allows the development of a more positive self-image, which is particularly lacking in burned-out parents and is fundamental for the therapeutic process. Indeed, the belief that one has the capacity to be a “good parent”—also called the parental self-efficacy belief—plays a key role in positive childrearing practices and satisfaction in one’s parental role [35]. On the one hand, self-compassion stimulates self-improvement [36], and on the other hand, it fosters parent–child attachment [37]. As a consequence, self-compassion training, along with mindfulness training, might help parents to implement new parenting skills and build positive and harmonious relationships with their children [38]. 

Surprisingly, a mindfulness and compassion-based approach to PB has scarcely been examined. Nevertheless, this interventional approach has shown beneficial effects on job burnout, highlighting the development of non-judgement and self-control skills as mediators [39,40]. Although this approach has been validated for parental stress (e.g., [41]), we cannot assert that it is as profitable for burned-out parents. Up to now, one study has investigated the effect of a mindfulness-based program (MBP) on PB. In line with the pioneering work by Lindström and colleagues [21] with parents of children with chronic illness, Anclair et al. [42] tested the effect of an MBP (derived from standardized mindfulness training programs) on stress and exhaustion in comparison to cognitive behavioral therapy. Beyond an early dropout rate of 18%, their study showed a large, positive impact of the MBP on exhaustion. Unfortunately, their results cannot be generalized to the population of burned-out parents. As a matter of fact, their sample was very specific (i.e., parents of children with chronic conditions), exhaustion was measured via a general instrument (not specific to parenting), and core facets of PB were not assessed (i.e., emotional distancing from one’s children and saturation with one’s parental role). Consequently, we cannot assert that their sample presented with *parental* burnout, or at least not with various forms of PBs (e.g., principally due to internal vs. external stressors), which may be associated with a different response to a MBP. With regard to the growing popularity of mindfulness in Western countries and the urgent need for PB treatment avenues, further investigation of mindfulness-based approach to PB seems timely.

Based on these considerations, the main aim of this study was to test the impact of an MBP, the mindfulness and compassion-based approach (MCA), in terms of parental burnout severity and associated outcomes. To accomplish this, we compared the impact of this treatment to the group treatment based on Brianda et al. [22], the Parenting in Balance Program (PBP), on multi-informant and multidimensional measures. Indeed, the literature on parental burnout has shown how informant-reported as well as physiological indices (i.e., hair cortisol), combined with self-reported measures, reflect the state of parental burnout [22,43,44]. The MCA structure was derived from standardized MBP with an adaptation to the population of burned-out parents who have limited attentional resources (i.e., shorter practices). While both interventions are grounded in the Balance between Risks and Resources framework of PB [3], the activities and language (i.e., reflecting mindfulness attitudes in the MCA) are distinct. Our objective was to isolate the effect of the approach to parental burnout by standardizing most characteristics of the interventions (i.e., group setting, sessions’ thematic, number, frequency and duration of sessions). Based on the literature, we hypothesized that both interventions would yield a more positive parental balance between stress-enhancing and stress-alleviating factors, as well as a decrease in parental burnout, parental neglect and violence, physiological stress, and irritability (H1). Due to a lack of data, we cannot assume the superiority of one intervention over the other. Our second hypothesis was that the MCA, more specifically, would increase mindful parenting and self-compassion (H2). We further hypothesized that the reduction in parental burnout would be associated with an enhancement of the parental balance between stress-enhancing and stress-alleviating factors in both interventions (H3), as well as that the reduction in parental burnout would be associated with an increase in mindful parenting and self-compassion in the MCA, more specifically (H4).

## 2. Materials and Methods

### 2.1. Participants

**Figure 1:**
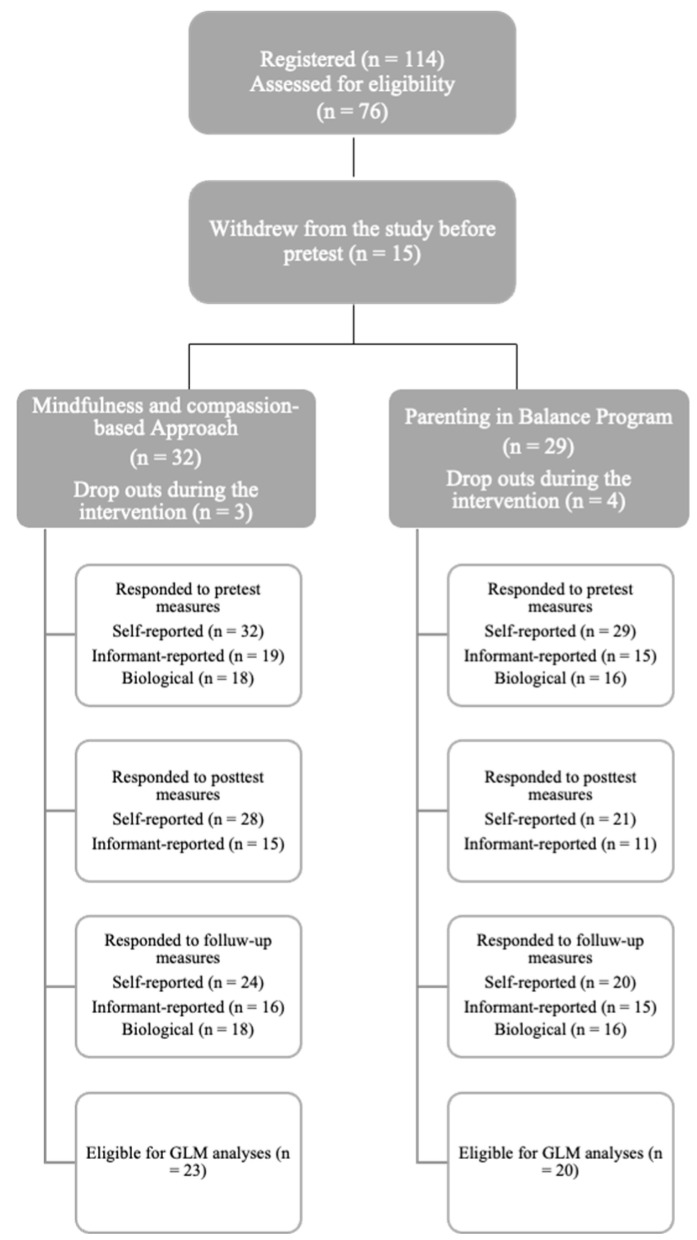
Flowchart diagram of participation rate at pretest, post-test, and follow-up measures.

G*Power calculation for a MANOVA repeated measures (within-between interaction) test indicated a minimum of 158 participants to detect a medium effect size (f = 0.25) with 80% power, such as that observed in previous interventional trials with burned-out parents [22,42]. From the 114 French-speaking parents who declared an interest in the study, a total of 76 participated in the study, out of which 54 parents (29 from the MCA, and 25 from the PBP) went through the whole intervention. Fifteen participants withdrew from the study before pretesting, and seven (three in the MCA, and four in the PBP) dropped out during the intervention. From the initial sample of parents assessed as eligible for the study, 44 (i.e., 28 in the MCA and 21 in the PBP) were included in the analyses as they responded to at least the pretest and post-test self-reported measures (see Figure 1 for flowchart diagram). The majority of participants were mothers (88%) aged between 35 and 39 (46%) (between 40 and 44: 25%), with two children (53%) (three children: 33%), living with a partner (73%), with higher education (92%), and working full-time (44%) (part-time: 23%). Please see Table 1 for a detailed presentation of the participants’ characteristics. Inclusion criteria were being over the age of 18 and having at least one child still living at home. Exclusion criteria specific to the MCA were the presence of psychotic symptoms, sequelae of physical or sexual abuse, and suicide risk, according to a previous mindfulness-based trial with stressed parents [41].

### 2.2. Measures

#### 2.2.1. Self-Reported Outcomes

Parental Burnout. Parental burnout was assessed using the Parental Burnout Assessment (PBA) [2]. This scale is composed of 23 items which measure common PB symptoms (e.g., I’m no longer able to show my children how much I love them). Responses are given using a 7-point frequency scale ranging from 0 (never) to 6 (every day). Therefore, the scores can vary from 0 to 138, with higher scores reflecting higher levels of PB. The clinical cutoff score is set at 75 [44]. In the current sample, Cronbach’s alpha was 0.97 at the pretest and post-test points, and 0.98 at follow-up.

Parental neglect. Parental neglect was assessed with the parental neglect scale [17], which is composed of 17 items measuring physical, educational, and emotional neglect (e.g., I don’t comfort my children when they are sad, frightened or distraught). Items are rated on an 8-point scale ranging from 0 (never) to 7 (several times a day). Therefore, total scores can vary from 0 to 119, with higher scores reflecting a higher frequency of neglectful behaviors. In the current sample, Cronbach’s alpha was 0.85 at the pretest, 0.79 at the post-test, and 0.80 at follow-up.

Parental violence. Parental violence was assessed using the Parental Violence Scale [17], which is a 15-item questionnaire encompassing verbal, physical, and psychological violence (e.g., I tell my children that I will abandon them if they are not good). Items are rated on an 8-point scale ranging from 0 (never) to 7 (several times a day). Therefore, total scores vary from 0 to 105, with higher scores reflecting higher frequencies of violent behaviors. In the current sample, Cronbach’s alpha was 0.87 at the pretest, 0.86 at the post-test, and 0.85 at follow-up.

Parental balance. The balance between stress-enhancing and stress-alleviating factors was assessed using the Balance between Risks and Resources (BR^2^) [3], a 39-item questionnaire that measures the multidimensional factors of parental burnout, including poor emotional regulation strategies and reduced co-parenting practices. The bipolar format of the items has the advantage of reflecting the notion of balance where resources are the opposite of risks (e.g., Left pole = Risk: My partner denigrates me as a mother/father. Right pole = Resource: My partner says that I am a good mother/father). Items are rated on an 11-point scale ranging from −5 (full endorsement of the risk factor) to +5 (full endorsement of the resource). In this case, 0 indicates that the parent has neither the risk factor nor the specific resource. The global score, computed by summing up the items, can vary from −195 to +195. Positive scores indicate that the parent has more resources than risk factors, while negative scores indicate that the parent has more risk factors than resources. A zero score means that the parent has the same level of risk factors and resources. Reliabilities were not computed for this measure since risks and resources are not necessarily expected to covary.

Mindful Parenting. Mindful parenting was assessed with the 10-item version of the Interpersonal Mindfulness in Parenting (IM-P) Scale [45]. This scale measures parents’ present-moment attention in parenting situations (e.g., I find myself listening to my child with one ear because I am busy doing or thinking about something else at the same time), present-moment emotional awareness in parenting (e.g., I notice how changes in my child’s mood affect my mood), non-reactivity/low-reactivity in parenting (e.g., When I’m upset with my child, I notice how I am feeling before I take action), and non-judgmental acceptance in parenting (e.g., I listen carefully to my child’s ideas, even when I disagree with them). Items are rated on a 5-point scale ranging from 1 (never true) to 5 (always true). The mindful parenting score is obtained by averaging the item scores; higher scores reflect higher levels of interpersonal mindfulness in parenting. In the current sample, Cronbach’s alpha of the translated version that we used (i.e., French translation by the first and third authors, back-translation by the fifth author) was 0.72 at the pretest, 0.57 at the post-test, and 0.59 at follow-up. As a measure of mindfulness is indispensable in such a study, we decided to keep IM-P score in our analyses despite insufficient psychometric properties.

Self-compassion. Self-compassion was assessed using the Unconditional Self-Kindness scale (USK) [46], which is a 6-item questionnaire measuring the extent to which one tends to react with tolerance and kindness in the face of difficult experiences (e.g., How much are you loving and kind to yourself when you fail or make a mistake?). Items are rated on a 7-point scale ranging from 0 (not at all) to 6 (strongly) and averaged to obtain a total score. Scores vary from 0 to 6, with higher scores reflecting a higher level of self-compassion. In the current sample, Cronbach’s alpha of the translated version that we used (i.e., French translation by the first and third authors, back-translation by the fifth author) was 0.92 at the pretest, 0.93 at the post-test, and 0.92 at follow-up.

#### 2.2.2. Informant-Reported Outcomes

Informant-reported PB. The partner’s or close other’s (in case there was no partner) perceptions of the participant’s PB symptoms were assessed using the Parental Burnout Assessment informant form (PBA-i), which is an adaptation of the PBA in which items of the PBA have been converted into a hetero-evaluation format (e.g., I have the impression that my partner is so tired out by his/her role as a parent that sleeping doesn’t seem like enough) [2]. It is designed to measure the partner’s perception of the parent’s level of burnout. In the current sample, Cronbach’s alpha was 0.97 at the pretest, 0.98 at the post-test, and 0.99 at follow-up.

Informant-reported irritability. The partner’s or close other’s (in case there was no partner) perception of the participant’s level of irritability was evaluated using an adaptation of the Carer’s Irritability Questionnaire (CIRQ) [47], which is a scale designed to measure a relative’s perception of irritable behaviors (e.g., The slightest thing puts him/her in a bad mood). The original instructions were adjusted for our purpose, and the respondents were asked to rate the frequency of their partner’s irritable behaviors using an 8-point scale ranging from never (0) to several times a day (7). The final version consisted of 15 items, with higher scores reflecting higher levels of irritability. Test scores were obtained by creating a mean score which varied from 0 to 7. In the current sample, Cronbach’s alpha was 0.91 at the pretest, and 0.95 at the post-test and follow-up.

#### 2.2.3. Biological Outcome

Hair cortisol. Hair cortisol levels, which provide an indication of chronic stress over the previous three months [24], were measured through hair samples of approximately 150 strands of hair collected from the posterior vertex of the head [48]. They were sent for analysis to a hair cortisol specialist at the Faculty of Pharmacy of the University of Granada. The cortisol in the hair sample was measured using the Salivary ELISA Cortisol kit© with the reagent provided, following the manufacturer’s directions (Alpco Diagnostics^®^, Windham, NH, USA). As the literature indicates high intraindividual stability of hair cortisol concentration [49], aside from high interindividual variance due to its numerous determinants (e.g., medication, hair color, hair washing frequency) [50], we calculated the percentages of change from the pretest to the follow-up for each participant separately.

**Procedure**

Parents willing to participate completed an online application form consisting of a brief screening questionnaire asking for their location of preference (6 cities from the French-speaking community in Belgium), their contact details for future communications, as well as inclusion and exclusion criteria. Cities were paired according to their average socio-economic levels beforehand in order to ensure that socio-economic background would not represent a confounding factor in the comparison between the two experimental conditions. Parents were blind to the numbers and types of conditions. The recruitment period started on 23 January and ended on 22 March 2019. Follow-up measures were collected between 16 September and 14 October 2019.

Eligible parents signed an informed consent form and completed a baseline questionnaire composed of sociodemographic items, as well as a brief 4-item measure of motivation (e.g., I feel ready to get involved in this intervention group). Thereupon, we invited participants by email to complete the assessment protocol four times via an online platform: one month before the beginning of the intervention (baseline), just before the beginning of the intervention (pretest), immediately after the end of the eight-week intervention (post-test), and three months after the end of the intervention (follow-up), basing their answers on the previous two weeks. Participants were invited to forward the link to the informant-reported questionnaire to their partner or a close other (an adult who saw them at least three times a week). Hair cortisol samples were collected at the beginning of the first session and the follow-up session in an adjacent room. In addition to outcome data at the post-test, participants from both interventions reported their attendance to each session (yes or no). Furthermore, participants from the MCA were asked to report the weekly average frequency (from 1 “less than once a week” to 3 “three or more times a week”) and duration (from 1 “a few minutes” to 4 “full length of the audio file”) of formal meditation, as well as the frequency (from 1 “less than once a week” to 3 “once a day or more”) of informal mindfulness practice at home, as it has been linked with MBP’s outcomes [51]. The design of the study was approved by the Institutional Review Board. As participants completed the baseline assessment at varying time distances from the pretest (i.e., between one month and a few days), data from this assessment period were removed from the study. The study protocol was not preregistered, as this was not a common practice in our labs at the time we prepared the trial, but is now registered in Open Science Framework (OSF) [52]. The authors confirm that all ongoing and related trials for this intervention are registered.

**Interventions**

Each group was led by one expert psychologist (lead instructor) assisted by a graduate student who was trained in PB treatment. Lead instructors of the MCA were clinical psychologists and doctors trained in mindfulness training and the implementation of mindfulness into psychotherapy, with parents or adults in general. Both interventions are grounded on the Balance between Risks and Resources framework [3], with the main goal of “restoring the balance” of participants by working on the most influential factors: social pressure on parenting (session 1), the identification of personal stressors and resources (session 2), parental perfectionism (session 3), emotional competencies (session 4), parent–child relationship quality (session 5), co-parenting (session 6), and help seeking (session 7). The closing session focused on relapse prevention.

**PBP**

PBP is a combination of the “Directive” and “Non-directive” interventions tested by Brianda et al. [22]. The main goal of the PBP, as in the “Directive” approach, was to lead participants to select the best ways to minimize stress-enhancing factors and maximize stress-relieving factors in their specific situations. In parallel, instructors helped parents to identify uncontrollable stressors in order to work on acceptance and avoid wasting what little energy they had trying to change the unchangeable. In line with the “Non-directive” approach, the PBP offered flexibility in the agenda of each session (adapting practices to expressed needs and difficulties) as well as periods of informal sharing between participants.

**MCA**

The MCA (see the Table 2 for an overview of the program) addressed factors from the parental balance via mindfulness and compassion-based practices in three ways. First, mindfulness training exercises (i.e., body scan, sitting meditation, yoga, and walking meditation) were introduced progressively through sessions, as in the Mindfulness-Based Stress Reduction program [53] and the Mindfulness-based Cognitive Therapy [54]. However, in-session and audio-guided home practices were shortened (i.e., 5 to 15 min) with respect to the level of parental fatigue. Second, several exercises from the Mindful Parenting training program [55] were included in sessions with related content (e.g., the morning stress exercise in session 2 on burnout etiology). Third, several exercises from the PBP were kept (e.g., detecting sources of pressure on parenting) and presented with a mindful attitude, following the steps of (1) experiencing through senses, (2) sharing lived experiences, and (3) elaborating on theoretical considerations and action plans. Other exercises were created for the MCA intervention, such as mental imagery on sharing parenthood.

### 2.3. Data Analysis

Statistical analyses were performed using IBM SPSS Statistics version 25. Significant outliers (3rd quartile ± 3×interquartile range) affecting the normality of distribution were identified through a boxplot analysis and suppressed (i.e., two aberrant BR^2^ scores, one at pretest and one post-test, as well as one extreme value for percentage of change of cortisol, within the PBP). As shown by one-way ANOVAs for discrete variables (i.e., motivation regarding the program, parental burnout, parental neglect and violence, parental balance, mindful parenting, self-compassion, informant-reported parental burnout and irritability, as well as number of children) and Pearson chi-square tests for categorical variables (i.e., sex, age category, marital situation, work regime, and level of education), the groups did not significantly differ from each other at pretest. Moreover, comparative analyses between individuals kept in the analyses (n = 28 from the MCA, and 21 from the PBP) and individuals who dropped out of the study after the pretest (n = 6 from the MCA and 8 from the PBP) showed no significant group differences as well, except for parental neglect, which was higher in individuals kept for analyses (*M* = 19.35, *SD* = 11.93) than in individuals whose data were removed (*M* = 11.69, *SD* = 10.91) (*F*(1, 59) = 4.36, *p* = 0.04, Cohen’s *d* = 0.53). Considering that attendance to sessions did not significantly differ between the MCA (*M* = 7.18, *SD* = 1.34) and the PBP (*M* = 7.62, *SD* = 0.59), and was not significantly related to parental burnout evolution from the pretest to the post-test, we did not include it in our analyses. 

To test our first and second hypotheses that both interventions would yield a more positive parental balance between stress-enhancing and stress-alleviating factors and a decrease in parental burnout, parental neglect and violence, physiological stress, and irritability (H1), as well as that the MCA, more specifically, would increase mindful parenting and self-compassion (H2), we ran generalized linear models for repeated measures, with time (pretest, post-test, and follow-up) as a within-subject factor and condition (MCA or PBP) as a between-subject factor, for each variable separately. Sphericity was checked via Mauchly’s test and the Greenhouse–Geisser correction was applied in case of violation. The percentage of changes in hair cortisol from pretest to follow-up was compared between groups via a one-way ANOVA.

To test our third and fourth hypotheses that the reduction in parental burnout would be associated with an improvement in the parental balance between stress-enhancing and stress-alleviating factors in both interventions (H3), as well as that the reduction in parental burnout would be associated with an increase in mindful parenting and self-compassion in the MCA (H4), we conducted correlation analyses on centered variables representing the differences in scores between the post-test and the pretest (∆ post-test–pretest) in (1) parental burnout, (2) parental balance, (3) mindful parenting, and (4) self-compassion. Since *p*-value depends on sample size, correlations were interpreted with reference to Cohen’s bounds (small, medium, large) [56].

As a complementary analysis, we checked whether the amount of home practicing had an impact on changes in mindful parenting and self-compassion from the pretest to the post-test via correlational analyses on centered data as well. Furthermore, in order to explore individual differences behind the reported means, we plotted parental burnout changes from the pretest to the post-test via a bar diagram for each condition.

## 3. Results

### 3.1. Variance Analyses

Generalized linear models for repeated measures did not reveal significant time*condition interaction effects. As shown in Table 3, however, all outcomes positively changed over time in both conditions, except for informant-reported irritability. Medium effect sizes were observed for parental balance and irritability (i.e., η^2^_p_ > 0.06), whereas large effect sizes were found for parental burnout (self- and informant-reported), parental neglect and violence, as well as mindful parenting and self-compassion (i.e., η^2^_p_ > 0.14). Importantly, participants reported sub-clinical cutoff (i.e., 86) levels of PB on average after the intervention in both conditions. As a corollary, both interventions led to a rise in the positive side of parental balance, although it was not maintained at follow-up in the MCA. However, a high standard deviation for the BR^2^ in this group indicates important individual differences.

Regarding hair cortisol concentration, the percentage of change from pretest to follow-up did not significantly differ between the MCA (*M* = 11.6, *SD* = 85.6) and the PBP (*M* = −28.91, *SD* = 46.51) (*F*(1, 32) = 2.72, *p* = 0.11). Again, a particularly high standard deviation in the MCA indicated important individual differences within that sub-sample.

### 3.2. Correlation Analyses

Correlation analyses on centered variables representing the difference in scores between the post-test and pretest did not outline significant relationships between parental burnout and potential mediating variables. With regard to H3, an augmentation of the parental balance (to the benefit of resources) tended to explain diminutions of parental burnout over the course of the PBP, with a large effect (*r* = −0.39, *p* = 0.09, *d* = 0.85), but yielded an opposite result for the MCA, with a medium effect (*rho* = 0.29, *p* = 0.14, *d* = 0.61). With regard to H4, the changes in parental burnout did not correlate with changes in mindful parenting (*r* = −0.13, *p* = 0.52, *d* = 0.26) or self-compassion (*r* = −0.08, *p* = 0.71, *d* = 0.16) over the course of the MCA.

### 3.3. Complementary Analyses

Pearson correlation analyses revealed that changes in mindful parenting from the pretest to the post-test were not significantly related to the frequency (*M* = 1.68, *SD* = 0.67) or mean duration of formal meditation practice (*M* = 2.39, *SD* = 1.34), nor the frequency of informal mindfulness practice (*M* = 1.82, *SD* = 0.67) at home (*r* = 0.17, *p* = 0.38, *d* = 0.35; *r* = −0.03, *p* = 0.87, *d* = 0.06; *r* = 0.29, *p* = 0.13, *d* = 0.61, respectively). Self-compassion evolution over the course of the intervention was not significantly related to the frequency (*r* = 0.32, *p* = 0.10, *d* = 0.68) or mean duration of formal meditation practice (*r* = −0.20, *p* = 0.32, *d* = 0.41), but was indeed correlated with the frequency of informal mindfulness practice at home to a large extent (*r* = 0.40, *p* = 0.03, *d* = 0.87).

**Figure 2:**
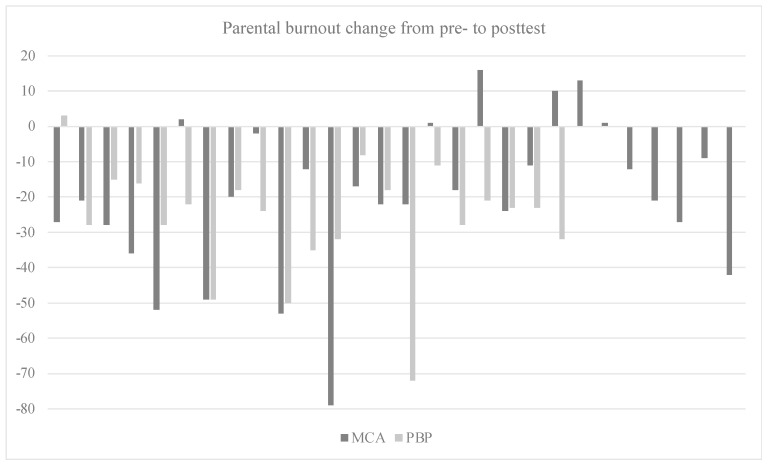
Bar diagram representing PBA individual score change from pretest to post-test.

As shown in Figure 2, six parents from the MCA and one parent from the PBP reported higher scores of parental burnout at the post-test than the pretest (+1, +1, +2, +10, +13, +16, and +3, respectively). While the bar diagram shows that most parents from the PBP benefited from the intervention (or did not undergo a worsening of their symptoms), it also highlights strong individual differences, with some parents thriving from the MCA and several others experiencing a deterioration in their levels of parental burnout (21% vs. 5% from the PBP).

## 4. Discussion

This study aimed to investigate the impact of a group mindfulness and compassion-based approach (MCA) regarding PB and its related outcomes in comparison to another group intervention based on previous research on PB [22], the Parenting in Balance Program (PBP). Overall, our results support the positive impact of the MCA and the PBP in terms of mean, but do not validate potential mediation mechanisms through mindful parenting or self-compassion, and unveil individual differences in the MCA that require further investigation for safe implementation with burned-out parents.

More precisely, our first hypothesis that both interventions would yield a more positive parental balance between stress-enhancing and stress-alleviating factors, as well as a decrease in parental burnout, parental neglect and violence, physiological stress, and irritability, was verified, except for the latter. Interestingly, informant-reported irritability was the only parenting-related outcome that did not significantly improve with the “Directive” and “Non-directive” approaches tested by Brianda et al. [22]. In terms of percentage of change, the two new approaches we assessed in our study showed similar patterns to the two validated ones. Nevertheless, the parental balance between stress-enhancing and stress-alleviating factors most strongly improved in the PBP (+31.27 points) in comparison with the “Directive” and “Non-directive approaches” (+15.42 points), as well as the MCA (+13.63 points), which further supports Brianda et al.’s argument for a hybrid approach to PB.

Surprisingly, our results did not fully support our second hypothesis that the MCA, more specifically, would increase mindful parenting and self-compassion. Indeed, these outcomes similarly increased with both interventions. Although we expected a specific increase in the MCA condition derived from formal mindfulness and self-compassion training, such a pattern was not highlighted by our data. Beyond the low adherence to home practices and the seemingly poor psychometric properties of the mindfulness scale, one possible explanation for the increases in mindful parenting in both conditions is that the interpersonal skills assessed by the IM-P, such as listening to one’s child and regulating one’s own emotions as a parent, were trained in both interventions through via different techniques (e.g., role play, relaxation, or meditation). Likewise, we may postulate that tolerance and patience towards one’s flaws or mistakes, as measured by the USK, was developed in both conditions through either common (e.g., the realization of shared vulnerability with other parents in both conditions) or different pathways (e.g., empathy during experience sharing, meditation). While explicit content and practices of self-compassion were found in the MCA only, both interventions offered the conditions for the development of a kind stance towards one’s suffering, through unconditional positive consideration and empathy, which was provided to all parents alongside the sessions. Nevertheless, as shown by our complementary analyses, the increase in self-compassion over the course of the MCA was related to the frequency of informal mindfulness practices at home, as previously outlined in the literature on MBP [57].

With regard to our third and fourth hypotheses that the reduction in parental burnout would be associated with an improvement in the parental balance between stress-enhancing and stress-alleviating factors in both interventions, as well as that the reduction in parental burnout would be associated with an increase in mindful parenting and self-compassion in the MCA, our results did not show any significant relationships. Although the fact that the increase in parental burnout was not related to an increase in mindful parenting nor self-compassion may be due to poor statistical power, this result challenges the theoretical assumptions behind the rationale of our study. An explanation for this null result, however, might be found in the exploration of moderating variables. For example, controlling for appreciation of mindfulness practices or personality traits related to mindfulness meditation practice [58,59] that increase dispositional mindfulness and self-compassion might reveal nuances in our findings. Moreover, exploratory analysis of individual data further revealed a deleterious effect of the MCA for several parents who reported heightened PB levels at the post-test. These observations exemplify the heterogeneity of cases behind mean intervention effects such as that in the sister field of job burnout [60], but also hold us back from fully endorsing a mindfulness-based approach to PB. Conversely, several individuals from the MCA condition substantially benefited from the intervention. Although preliminary, this result calls for reflection on the way in which mindfulness practices are proposed to burned-out parents. Indeed, although mindfulness training may address core dysfunctional processes within burnout, mindfulness training may not be appropriate for everyone or at every phase of the syndrome. In line with other MBPs (e.g., depression relapse prevention; [54]), we believe that MCA for PB may be most appropriate when parents partially overcome their distress and recover a feeling of “safety” within themselves (i.e., soothing-affect systems; [33]), in order for meditation practice not to be too distressing or overwhelming. Most importantly, participants in the MCA condition did not initially choose mindfulness as a treatment method, as opposed to most studies on MBP. As a consequence, we may postulate that the positive effects we found might have been increased if parents had voluntarily engaged in an MBP such as the MCA. Indeed, while the blindness of the participants to the treatment conditions was one of the strengths of this study, and allowed us to control for group biases (e.g., with a specific profile of parents engaging in mindfulness practice), this might have elicited feelings of surprise and rejection in people reluctant to practice meditation and impeded the therapeutic process.

Overall, the absence of clear distinctions between the MCA and the PBP effects suggests the importance of common factors between conditions, such as the group setting and the session topics (i.e., parenting-related stress-alleviating factors), as explanatory factors. Interestingly, Anclair et al. [42] found that group treatment for parents of children with chronic conditions, with either CBT techniques or a mindfulness-based program, effectively reduced stress and burnout symptoms. Brianda et al. [22], for their part, also failed to observe interactions between the time and condition of PB treatment for any outcome, further demonstrating the relative insignificance of the technique being used (i.e., mindfulness or other).

### Limitations and Future Research

Although our study yielded encouraging results, it also bears several limitations that require caution in the interpretation of the results. One of the most important is its insufficient sample size with regard to statistical power. Indeed, although we recruited all the parents who expressed an interest, this did not enable us to reach a sufficient number of parents to satisfactorily test all our hypotheses.

Another limitation relates to the comparison of our interventions. Indeed, the comparison of our interventions would have been more informative if other potential mediating variables (e.g., feelings of shame and guilt, self-efficacy, autopilot mode in general) had been taken into account in our statistical models. Indeed, although the outcomes were similar across interventions, the mechanisms by which they occurred may have differed according to the approach (e.g., reduction of the auto-pilot mode in the MCA condition). Future interventional studies should therefore explore the role of a broader set of mediating variables in order to better explain and predict outcomes associated with PB treatments. Beyond the two approaches to PB that we chose to test, our study lacked an inactive group that would have allowed us to control for the effect of time on the evolution of symptoms following intervention. However, data from a waiting-list sample in the interventional study on PB led by Urbanowicz et al. [20] showed no significant effect of time for any of the outcome variables.

Finally, further efforts should be made in the investigation of individual differences, such as the role of other moderating variables (e.g., comorbidities, partner’s implication in the therapeutic process, a priori preference for treatment type), as encouraged by the literature on MBP and burnout [60]. Indeed, MCA was very helpful for a proportion of our sample, and we need to identify their profiles, as well as those of individuals who negatively responded to that approach. In conclusion, the variety in individual trajectories which we graphically observed within both conditions highlights the amplitude of the work that remains to be conducted in the clinical field of PB and beyond, and warns both researchers and clinicians about the challenges of using MBP to treat parental burnout. This study thus represents a new contribution on theoretical and practical levels. Indeed, on a theoretical level, this study highlights common intervention components that lead to reduced parental burnout through parenting support groups that increase self-kindness. On a practical level, a major implication relates to the importance of considering parents’ profiles in order to find the best fit for their motivation, difficulties, and needs, as well as the type of program proposed as suggested by past works on general well-being enhancement interventions [60] (see the person–activity fit model by Lyubomirsky and Layous, 2013). Furthermore, in line with past studies on PB reduction programs [22], this study highlights the relevance of such parenting support interventions during important crisis periods, such as the COVID-19 pandemic, which increased the risk for parental burnout [11]. 

## Figures and Tables

**Table 1:** Participants’ sociodemographic characteristics.

Characteristic		*N* (%) ^1^		
		MCA	PBP	Total
Sex	Men	3 (10.7)	3 (15)	6 (12.5)
	Women	25 (89.3)	17 (85)	42 (87.5)
Age category	30–34 years	5 (17.9)	2 (10)	7 (14.6)
	35–39 years	12 (42.9)	10 (50)	22 (45.8)
	40–44 years	6 (21.4)	6 (30)	12 (25)
	45–49 years	3 (10.7)	1 (5)	4 (8.3)
	50–54 years	1 (3.6)	1 (5)	2 (4.2)
	55–59 years	1 (3.6)	0	1 (2.1)
Number of children	1	2 (7.1)	1 (4.8)	3 (6.1)
	2	16 (57.1)	10 (47.6)	26 (53.1)
	3	6 (21.4)	10 (47.6)	16 (32.7)
	4	3 (10.7)	0	3 (6.1)
	5	1 (3.6)	0	1 (2)
Marital status	Living with a partner	20 (71.4)	15 (75)	35 (72.9)
	Solo parent	8 (28.6)	5 (25)	13 (27.1)
Level of education	Secondary	2 (7.1)	2 (10)	4 (8.3)
	Bachelor’s or master’s	23 (82.1)	14 (70)	37 (77.1)
	Doctoral or equivalent	3 (10.7)	4 (20)	7 (14.6)
Work regime	Full-time	14 (50)	7 (35)	21 (43.8)
	Half-time	1 (3.6)	4 (20)	5 (10.4)
	Part-time	6 (21.4)	5 (25)	11 (22.9)
	Unemployed	3 (10.7)	3 (15)	6 (12.5)
	Inability to work	3 (10.7)	1 (5)	4 (8.3)
	Stay-at-home parent	1 (3.6)	0	1 (2.1)

^1^ All sociodemographic characteristics except the number of children are lacking for one participant within the PBP condition.

**Table 2:** Overview of the MCA program.

Session	Exercises
1. Being a parent in the 21st century	Presentations; raisin exercise; detecting idealization *Child as a raisin* ^1^*; detecting pressure for perfect parenting; mindful breathing*
2. Each burnout has its own story	Body scan; morning stress exercise ^1^; list of depleting/resourcing activities *Body scan; calendar of positive experiences*
3. About parental perfectionism	Yoga/stretching; mental imagery on overinvestment; bringing kindness to yourself ^1^ *Yoga/stretching; breathing space; calendar of negative experiences*
4. Developing emotional competencies	Mental imagery on guilt; meditation on the breath and body *Meditation on the breath and body; breathing space in difficult times; calendar of reactions/responses*
5. Revaluing the relationship with children	Mindful walking; meditation on sounds and thoughts; imagination exercise: Limits ^1^ *Meditation on sounds and thoughts or yoga; calendar of child’s positive emotions; mindful quality time with child*
6. The parental team	Imagination exercise: sharing parenthood; perspective taking in conflict situations; active listening *Sitting meditation or yoga; calendar of coparental interactions*
7. Asking for help	Mental imagery on help seeking; chocolate exercise (awareness of interdependency); non-violent communication *Chosen meditation; calendar of help requests; encouraging children’s autonomy*
8. Preventing relapse	3-walk exercise; balance exercise; discussion on follow-up

^1^ Exercises retrieved from the Mindful Parenting program [55]. Home practices are italicized.

**Table 3:** Means and standard deviations at each measurement time, time effect, partial eta-squared, and percentage of change from pretest to follow-up for each dependent variable.

		Pretest	Post-Test	Follow-Up	% of Change	*F*(df) *	η^2^_p_
PBA	MCA	90.83 (30.32)	70.12 (30.09)	65.46 (35)	−28%	34.74 (1.76, 73.78)	0.45
	PBP	87.2 (31.66)	60.85 (28.24)	57.35 (32.85)	−30%		
Neglect	MCA	20.79 (11.89)	17.13 (11.2)	14.21 (8.92)	−32%	11.99 (2, 84)	0.22
	PBP	19.65 (12.33)	14.9 (6.75)	14.5 (9.55)	−26%		
Violence	MCA	16.09 (8.32)	12.13 (7.66)	11.52 (7.42)	−28%	14.88 (1.59, 63.54)	0.27
	PBP	17.95 (11.95)	11.37 (6.98)	11.84 (7.12)	−34%		
PBA-i	MCA	79 (39.23)	61.25 (29.89)	66 (37.7)	−16%	9.87 (2, 36)	0.35
	PBP	94.38 (31.26)	77.63 (42.48)	62.38 (38.26)	−34%		
IRR-i	MCA	2.52 (1.35)	2.23 (1.53)	2.43 (1.48)	−4%	2.73 (2, 36)	0.13
	PBP	3.23 (1.07)	2.72 (1.26)	2.32 (1.35)	−28%		
BR^2^	MCA	−19.71 (48.2)	0.79 (48.72)	−6.08 (55.13)	+13.63 ^b^	4.98 (1.67, 68.6)	0.11
	PBP	−0.95 (23.89)	15.84 (31.24)	30.32 (31.01)	+31.27 ^b^		
IM-P	MCA	3.13 (0.47)	3.42 (0.39)	3.31 (0.4)	+6%	12.98 (1.73, 72.67)	0.24
	PBP	3.11 (0.33)	3.34 (0.25)	3.37 (0.23)	+8%		
USK	MCA	2.95 (0.98)	3.66 (1.2)	3.55 (1.4)	+20%	16.22 (2, 84)	0.28
	PBP	2.68 (1.09)	3.47 (1.12)	3.84 (0.73)	+43%		

* All *p*-values are < 0.001 except for BR^2^ (*p* = 0.01) and IRR-I (*p* = 0.08). ^b^ Score difference. PBA = Parental Burnout Assessment; Neglect = Parental Neglect scale; Violence = Parental Violence scale; PBA-I = Parental Burnout Assessment informant form; IRR-I = Carer’s Irritability Questionnaire–adjusted informant form; BR^2^ = Balance between Risks and Resources; IM-P = Interpersonal Mindfulness in Parenting; USK = Unconditional Self-Kindness scale.

## Data Availability

The data presented in this study are openly available in OSF at 10.17605/OSF.IO/R2N3H.
